# Acute myeloid leukemia xenograft success prediction: Saving time

**DOI:** 10.1016/j.exphem.2017.12.002

**Published:** 2018-03

**Authors:** Emmanuel Griessinger, Jacques Vargaftig, Stuart Horswell, David C. Taussig, John Gribben, Dominique Bonnet

**Affiliations:** a INSERM U1065, C3M, Team 4 Inflammation, Cancer, Cancer Stem Cells, Nice, France; b Haematopoietic Stem Cell Laboratory, The Francis Crick Institute, London, United Kingdom; c Division of Hematology, René Huguenin Hospital—Curie Institute, Saint-Cloud, France; d Royal Marsden Hospital, Sutton, Surrey, United Kingdom; e Institute of Cancer Research, Sutton, United Kingdom; f Department of Haemato-Oncology, Barts Cancer Institute, Queen Mary University of London, London, United Kingdom

## Abstract

Xenograft assay allows functional analysis of leukemia-initiating cells of acute myeloid leukemia primary samples. However, 40% of samples derived from patients with better outcomes fail to engraft in immunodeficient mouse recipients when conventional protocols are followed. At diagnosis, the engraftment of intermediate-risk group samples cannot be anticipated. In this study, we decided to further explore the reasons for xenograft success and failure. No differences in extracellular phenotype, apoptosis, or cell cycle profile could distinguish samples that engraft (engrafter [E]) from samples that do not engraft (nonengrafter [NE]) in NSG mice. In addition, ex vivo long-term culture assay revealed, after 5 weeks, a lower content of leukemic-LTC-initiating cells in the NE samples associated with a lower expansion rate capacity. One-week co-cultures with mesenchymal or osteoblastic or endothelial cells did not influence the proliferation rate, suggesting that E and NE samples are genuinely rapidly or slowly expanding independent of external cue. Engraftment success for some NE samples was consistently observed in recipient mice analyzed 6 months later than the conventional 3-month period. Eventually we implemented a flow cytometry-based assay, which allowed us to predict, in 1 week, the fast or delayed engraftment potential of a noncharacterized acute myeloid leukemia sample. This approach will be especially useful in selecting intermediate-risk-group patient samples and restricting the experimental duration to a 3-month period and, eventually, in reducing the number of animals and the cost and effort of unnecessary xenograft failures.

## HIGHLIGHTS

• Extracellular phenotype, apoptosis, or cell cycle profile at thawing cannot predict acute myeloid leukemia (AML) xenograft potential.
• Leukemic long-term culture-initiating cell content distinguishes fast and delayed engraftment potential.
• Defined here is a 1-week carboxyfluorescein succinimidyl ester-based assay that predicts AML xenograft success.

The in vivo xenotransplantation assay in NOD/SCID IL-2Rγ common chain null (NSG) mice is currently the model most frequently used to study the biology of leukemia-initiating cells (LICs); however, a substantial proportion of samples from patients with acute myeloid leukemia (AML) with a good prognosis fail to engraft in mice. Other newly described humanized mouse models such as NSG-SG3M and MISRTG mice might improve such sample engraftment [1]. Yet, we recently evidenced the extinction of myelodysplastic syndrome propagating cells (MDS-PCs) using NSG-SG3M mice, which suggests that human cytokine stimulation might exhaust the LIC compartment of particular leukemias [2]. Alternatively, we found that subcutaneous implantation of gelatin sponges seeded with human stromal cells allows engraftment of good-risk AML in NSG mice. However, as observed by others using subcutaneous humanized ossicles, these ectopic leukemic grafts do not invade recipient bone marrow 3, 4, 5. Because all these models are either not fully characterized or not fully available, the straightforward intravenous NSG model is still the most commonly used model. Here we further investigated xenograft failure in this model and developed a flow cytometry-based assay that allow prediction of the xenograft potential of a noncharacterized AML sample.

## Methods

### Cells

AML cells were obtained after receipt of informed consent from St Bartholomew's Hospital. Details of the patient samples are listed in Supplementary Table E1 (online only, available at www.exphem.org). Co-culture experiments were previously described [6]. AML samples were collected at diagnosis, and mononucleated cells were isolated within 24 hours after collection by Ficoll-Paque Plus density gradient (GE Healthcare, France). Cord blood (CB) cells were obtained after receipt of informed consent from the Royal Free Hospital (UK). Both AML and CB sample collections were approved by the East London ethical committee and in accordance with the Declaration of Helsinki. Three to 5 different CB samples were pooled, and mononuclear cells were obtained by density centrifugation. Lineage markers expressing cells were depleted using StemSep columns and human progenitor enrichment cocktail (StemCell Technologies, Vancouver, BC, Canada). CD34^+^CD38^−^ cells (hematopoietic stem progenitor cells [HSPCs]) and CD34^+^CD38^+^ cells (hematopoietic progenitor cells [HPCs]) were sorted on a MoFlo cell sorter (DakoCytomation Colorado, Fort Collins, CO, USA) or a BD FACS Aria (BD Biosciences, UK). Gates were set up to exclude nonviable cells and debris. Briefly, lineage-depleted recovered cells were washed twice and stained with anti-CD34 Percp, anti-CD38 PE-cy7, AlexaFluor647-conjugated Annexin-V (Invitrogen), and DAPI (4',6-diamidino-2-phenylindole). The purity of sorted fractions was assessed to ensure the sort quality. The stromal cell line mesenchymal MS-5 and the human osteosarcoma cell line SaOS-2 were obtained from the DSMZ cell bank (Braunschweig Germany) and maintained in Iscove's modified Dulbecco's medium (IMDM) containing 10% fetal calf serum (FCS) + 2 mmol/L L-glutamine or in McCoy's 5a medium containing 15% FCS + 2 mmol/L L-glutamine, respectively. Human umbilical vein endothelial cells (HUVECs) obtained from Lonza were propagated in endothelial growth medium-2, EGM-2-MV (Lonza, UK) in culture dishes coated with type I collagen (StemCell Technologies). MS-5, SaOS-2, and HUVEC feeders were cultured in their respective media and subcultured when reaching 80% confluence. Sca-1, CD56, and CD31 were identified as specific markers for 100% of MS-5, SaOS-2, and HUVEC, respectively, and used for feeder exclusion in fluorescence-activated cell sorting (FACS) analysis. All three antibodies were from BD Pharmingen (Oxford Science Park, UK).

### Adoptive transfer of human hematopoietic cells in immunodeficient mice

**Supplementary Figure E1:**
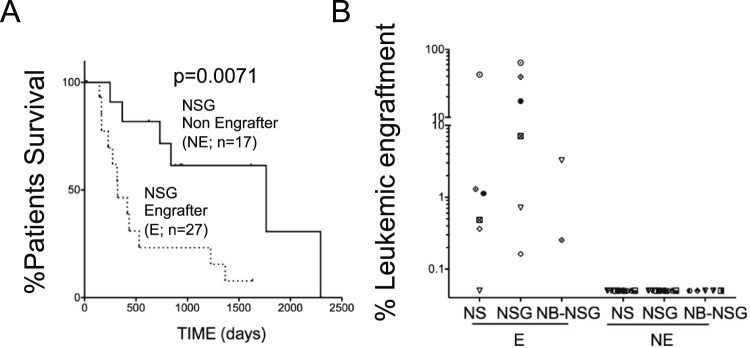
Better outcome AML cannot engraft adult and newborn NSG mice. **(A)** Patients with AML cells devoid of xenograft potential in NSG mice (n = 17) have better overall survival than engrafter AML (n = 27). Data show Kaplan-Meier 6 years survival curves of de novo AML cases (<60 years old) that received intensive multi-agent chemotherapy. Allografted patients were censored. *p* = 0.0071 in a Mantel-Cox log rank test. **(B)** Data show the percentage of engraftment of engrafter (E) and non engrafter (NE) in NOD/SCID (NS) versus NOD/SCID IL-2Rγ common chain null (NSG) or newborn NSG mice (NB-NSG) models. 11 NS NE sample were tested in the NS and NSG model, of which 5 in the NB-NSG model. 6 NS E were tested in the NS and NSG model of which 2 in the NB-NSG model. 1–10 10^6^ T cells depleted mononuclear cells were injected and the leukemic engraftment level was evaluated by FACS at 10 to 12 weeks. Each dot represents the mean engraftment level of a single AML sample determined for n = 2 to 12 animals per sample.

NOD/SCID (NS) and NSG mice were a kind gift of Dr. Leonard Shultz. All animal experiments were performed in accordance with Home Office and CRUK guidelines. Adult NS or NSG mice were injected intravenously with 10^7^ T-depleted mononuclear AML cells. In the current study, we define nonengrafter [NE] samples as samples for which 10^7^ CD3^+^-depleted AML MNCs injected cells were not able to engraft at a detectable level (cutoff: 0.1%) of human myeloid-only leukemic population CD45^+^CD33^+^CD19^−^ and murine CD45^−^, 12 weeks after injection into NSG mice. For newborn xenograft, 2.5 to 10 × 10^6^ AML cells were injected into day 2 neonate NSG mice via an intrahepatic (IH) or facial (FV) vein. For some engrafter (E) samples, LIC and non-LIC phenotypes were functionally defined by xenograft experiments with sorted subpopulations (Supplementary Figure E1, online only, available at www.exphem.org). Mouse bone marrow cells were collected and analyzed by flow cytometry as detailed previously 7, 8.

### Long-term culture

Co-culture experiments were performed as previously described 9, 10 as bulk culture or using a limiting dilution analysis (LDA) both on confluent monolayers of MS-5, supplemented with recombinant human interleukin (IL)-3, granulocyte colony-stimulating factor (G-CSF), and TPO (MS-5 + 3GT) (20 ng/ml each; Peprotech, London, U.K., http://www.peprotech.com) in MyeloCult H5100 (StemCell Technologies, Vancouver, BC, Canada, http://www.stemcell.com). Cells were cultured at 37°C in 5% CO_2_-humidified incubators. Cells were plated in 20 replicates in 96-well microplates containing confluent MS-5 monolayers. Half the medium was done twice a week without disrupting the established feeders. After 5 weeks, LTC medium was replaced by methylcellulose H4435 (StemCell Technologies). After an additional 2 weeks, each well was scored as negative if no colonies were present. To determine the frequency of leukemia long-term culture-initiating cells (L-LTC-ICs), LDA was calculated using LCalc software (StemCell Technologies) according to Poisson statistics and the method of maximum likelihood.

### Fluorescence dilution factor

AML cells (1 to 10 × 10^5^) were thawed and stained with 0.8 µmol/L carboxyfluorescein diacetate succinimidyl ester (CFSE) (Invitrogen, UK) for 10 min at 37°C in phosphate-buffered saline (PBS). Cells were washed twice and incubated in duplicate on pre-established confluent MS-5 (or SAOS-2 or HUVEC) layers at 37°C in Myelocult medium (StemCell Technologies) without cytokine supplementation. Cells were incubated 18 hours before assessing the initial input CFSE fluorescence intensity to allow for the turnover of CFSE-labeled proteins to stabilize [11]. After 18 hours and 1 week, wells were harvested by trypsinization and stained with anti-Sca-1-PE (for MS-5 staining), or anti-CD56-PE (for SaOS-2 staining), or anti-CD31-PE (for HUVEC staining) and anti-CD45-APC-Cy7 antibodies (BD Pharmingen). The CFSE median fluorescence index (MFI) was measured by FACS at 18 hours and day 7 on viable (Annexin V and DAPI negative) human hematopoietic cells (CD45 positive and Sca-1 negative). FDF was defined as the ratio of the 18-hour CFSE MFI to the 1-week CFSE MFI. AML heterogeneity evidenced through FACS scattered light measurements meant that the width of the labeled input population exceeded the limits for peak resolution, even under optimal instrument conditions 12, 13. As we reported previously [10], larger cells labeled more brightly with CFSE compared with smaller blasts within the same sample. Consequently, as the cells divide, the width of each division peak overlaps heavily with previous and subsequent peaks, preventing accurate peak resolution. Instead, we defined the fluorescence dilution factor (FDF) as the ratio of the 18-hour CFSE MFI to that of the D7 CFSE MFI. The CFSE MFI was measured on viable (Annexin V-Alexa Fluor 647 and DAPI negative) human hematopoietic cells (CD45-APC-Cy7 positive and Sca-1-PE or CD56-PE or CD31-PE negative, excluding residual normal lymphocytes CD45^high^/SCC^low^ for all analyses. At day 7, the same procedure was applied. Cytometric calibration was controlled using CountBright beads.

### Flow cytometry analysis

FACS analysis was performed with a BD LSRII flow cytometer (BD Biosciences, UK). After 1 to 5 weeks of co-culture, nonadherent and adherent cells were harvested through trypsinization. Recovered cells were resuspended and stained in Annexin binding buffer (BD Biosciences) and with anti-SCA-1-PE or anti-CD56-PE or anti-CD31-PE, and anti-CD45-APC-Cy7, anti-CD34-Percp, anti-CD38-PE-Cy7 antibodies, and Lin-FITC (BD Biosciences), as well as with AlexaFluor647-conjugated Annexin-V (Invitrogen) and DAPI (Sigma). Only viable (both DAPI and Annexin-V negative fraction) human hematopoietic cells (CD45-APC-Cy7 positive and Sca-1-PE or CD56-PE or CD31-PE negative) were assessed for all analyses. Cell viability was expressed as the percentage of DAPI- and Annexin-V-negative cells within human hematopoietic cells.

#### Cell cycle analysis

Cells were fixed in 2% paraformaldehyde for 10 min, permeabilized with 0.1% Triton X-100 for 10 min, resuspended in 500 µL of PBS with 2% FCS and 1 µg/mL DAPI for 15 minutes, and processed for cell cycle analysis on an LSRII analyzer (BD Pharmingen). Cells in G1 were those with 2N DNA content and Ki-67-positive cells, cells in S were those with a DNA content ranging from 2N to 4N, and cells in G2/M were those with a 4N DNA content.

#### Fold expansion

After 1 to 5 weeks of co-culture, well content was harvested by trypsinization and stained, and CountBright absolute counting beads were used to assess by FACS the total number of viable human hematopoietic cells following the manufacturer's recommendations. For LTC, no accumulative count was applied because hematopoietic cell adhesions vary from one feeder to another and no precise semipopulation could be operated.

### Statistics

Data were analyzed for statistical significance using the nonparametric unpaired Mann–Whitney or paired two-tail test. The Kaplan–Meier method was used to establish patients' overall survival curves, and the Mantel–Cox log rank test was used to assign a significance level. Observed differences were regarded as statistically significant if the calculated two-sided *p* value was <0.05.

### Mathematical model

Engraftment status was encoded as a binary variable and a logistic predictive model was built for this variable based on the interaction of FDF and viability. More precisely, R function generalized linear models (GLMs) was used to fit a logistic model with family binomial and predictive formulas. Engraftment status = viability + FDF/viability. The test reveals that use of the full model (i.e., including a separate index term) did not have a significant effect on the resultant model (as assessed by the R function analysis of variance) and so was omitted. The empirical false detection rate for various predictive thresholds was calculated by explicitly calculating the number of false predictions made when applying the model to the observed data. All calculations were performed using R 3.0.1.

## Results and Discussion

**Supplementary Figure E2:**
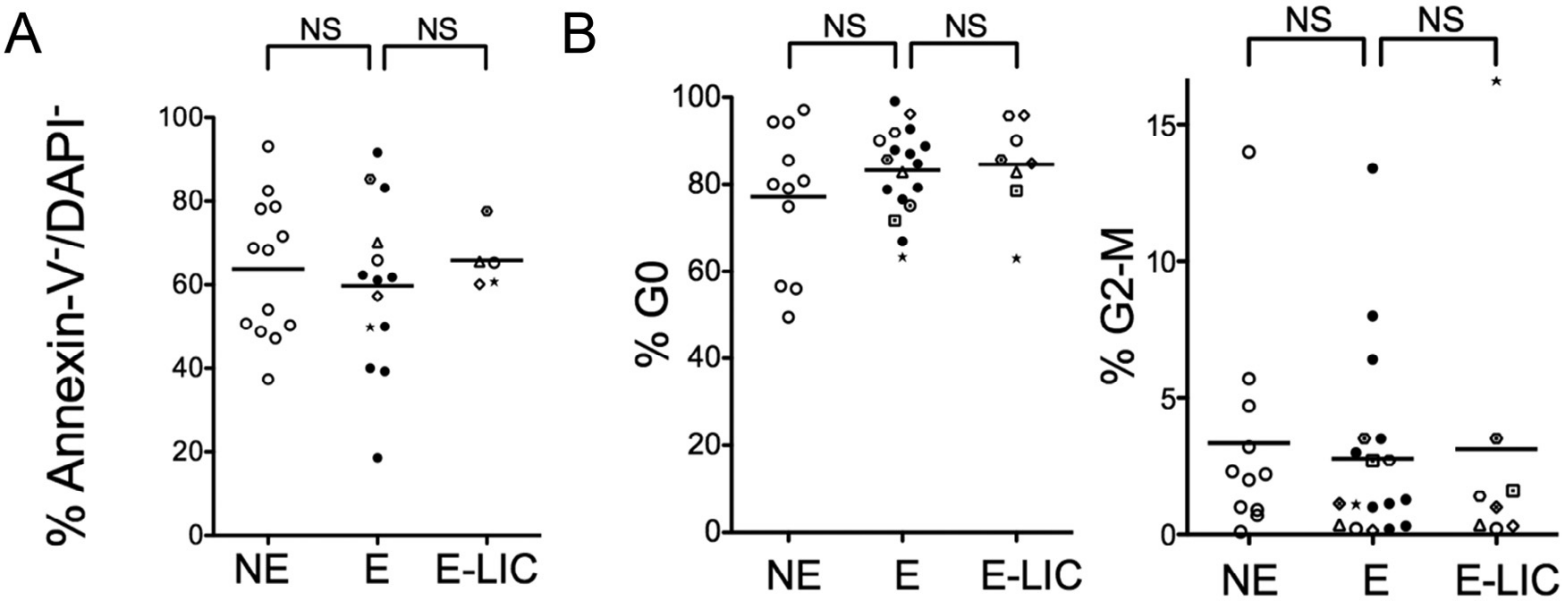
Cell viability or cell cycle profiling at thawing do not account for engraftment defect. **(A)** Data shown represent the post-thaw AML percent cell viability assessed by DAPI and Annexin V exclusion for Non Engrafter (NE, open symbols), Engrafter (E, closed and single symbols) and Engrafter LIC subpopulation (E-LIC) (n = 13, 14 and 5 samples respectively). **(B)** Cell-cycle profile determined by Ki67 and DAPI staining for NE, E and E-LIC (n = 11, 18 and 8 samples respectively). Plain lines represent mean levels in each group. A single symbol is used for E and E-LIC matched samples. For few samples the LIC and non-LIC phenotype were first functionally pre-established through xenograft experiments on sorted sub-population based on CD34 and CD38 expression. No significant differences were identified between the different groups in parametric or non-parametric paired or unpaired tests.

**Supplementary Figure E3:**
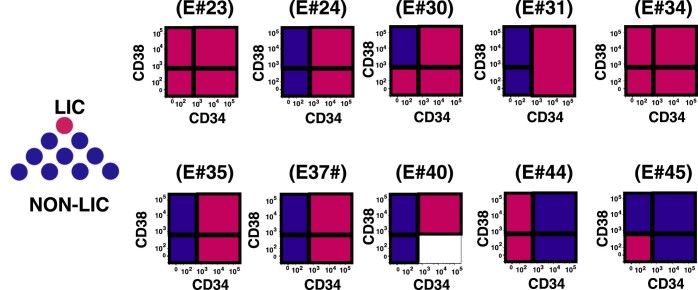
Schematic LICs (red) and NON-LIC (blue) phenotype repartition based on CD34 CD38 for 10 AML samples. AML cells were sorted based on the CD34 and CD38 expression and injected in NSG mice (n = 3 to 8 animals per gate tested). Fraction was scored as enclosing LICs if a distinct human single CD45+CD33+CD19− population was detectable in the recipient bone marrow. For E#40 CD34+CD38− quadrant was not tested since no cells harbored this phenotype.

We analyzed 45 AML samples, 18 of which (40%) could not give rise to leukemic engraftment when injected intravenously into NOD/SCID mice (NS), β_2_-microglobulin-deficient (B2m(null)) NS or NSG mice 8, 14 (Supplementary Table E1, online only, available at www.exphem.org). As reported before by our group [8], we confirm, in this new cohort of patients, a strong negative correlation between potential and patient survival (Supplementary Figure E2A). The newborn NSG model, although described as an even more permissive xenograft assay [15], revealed itself helpless because none of the five nonengrafting (NE) samples tested in this model produced a detectable graft (Supplementary Figure E2B). We first wondered whether intrinsic differences between NE and engrafter (E) mice might be identified to explain engraftment success or failure. We tested the hypothesis of a lower frequency of LICs in the NE samples; however, no differences were observed phenotypically in terms of the proportion of CD34^+^ or CD34^+^CD38^−^ cells when comparing 27 E and 18 NE mice (Supplementary Table E1). Also, similar heterogeneous viability and a similar mean percentage of cells in the G0 or G2/M phases were quantified in both the E and NE groups (Supplementary Figure E1). We questioned if analyzing the bulk AML population might conceal the actual LIC signal. To test this hypothesis, we compared apoptosis level and cell cycle profile in the same sample, between LICs and bulk AML of E samples. LIC phenotype was determined for 10 E samples by assessing the xenograft potential of different sorted fractions (Supplementary Table E1; Supplementary Figure E3, online only, available at www.exphem.org). Heterogeneous LIC phenotype was observed, confirming already published data 7, 16, 17. Two samples were even censored because xenograft potential was found in all cytometry quadrants defined by CD34 and CD38 expression. For the 8 remaining samples, the apoptosis level or cell cycle profile was very similar for the LIC enclosing sub-compartment and the entire sample population, suggesting those parameters are surprisingly shared between LICs and the non-LICs (Supplementary Figure E1). When combined, these data suggested that cell phenotype, viability, or cell cycle profiling markers after thawing cannot account for the engraftment defect in NE samples.

**Figure 1:**
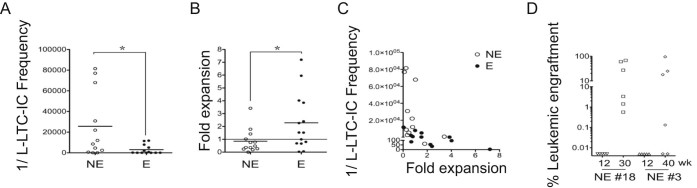
NE AML samples have a lower L-LTC-IC compartment size and a lower long-term expansion rate. **(A)** L-LTC-IC frequencies measured for 12 NE (*open symbol*) and 13 E (*closed symbol*). Data shown represent for each sample the initial cell number necessary for leukemic long-term culture maintenance (1/L-LTC-IC frequency). Plain lines represent mean levels in each group. **(B)** Data shown represent the fold expansion of 13 NE and 14 E samples after 5 weeks of co-culture on MS-5. Each symbol represents the mean result obtained from a single AML sample cultured in 5 to 20 wells. For **(C)** and **(D)**, comparisons were made using a Mann–Whitney *t* test. *p < 0.05. **(C)** Fold expansion in function of the respective L-LTC-IC frequency. **(D)** Percentage engraftment in NSG mice of two NE: Nos. 18 and 3 at 12 and 30 and 12 and 40 weeks after injection, respectively. AML = acute myeloid leukemia; E = engrafter; L-LTC-IC = leukemic long-term culture-initiating cells; NE = nonengrafter.

We recently determined that good-risk AML patient samples at diagnosis have a five to seven times lower frequency of leukemic long-term culture-initiating cells (L-LTC-ICs) compared with intermediate- and poor-risk AML samples [10]. Long-term cultures revealed that NE samples also have a lower content of L-LTC-ICs. Consequently, 8.41 times more NE cells were required at day 0 to obtain positive wells at week 5 as compared with E samples (Fig. 1A, *p* < 0.05). NE samples also yielded, on average, 2.7 times fewer leukemic cells than E samples at week 5 (Fig. 1B, *p* < 0.05). Plotting L-LTC-IC frequency against fold expansion, determined at week 5, highlighted the tight relationship between these two parameters and allowed differentiation of the low-expanding, L-LTC-IC-impoverished NE samples from the high-expanding, L-LTC-IC-enriched E samples (Fig. 1C). Favorable-risk leukemic cells were recently reported to require up to 1 year to establish a detectable graft in recipients [6]. Effectively, two NE mice that failed to engraft in NS or NSG mice at 12 weeks were also investigated at 30 or 40 weeks. For the two samples, a distinct leukemic engraftment was seen at weeks 30 and 40, respectively (Fig. 1D). Thus, conventional intravenous inoculation distinguishes short- and long-latency engrafter mice, rather than discern absolute engraftment potential. However, we cannot exclude the acquisition of an addition mutation(s) over this duration nor that the leukemic cells might have modified the bone marrow microenvironment to reach leukemia onset 18, 19.

**Figure 2:**
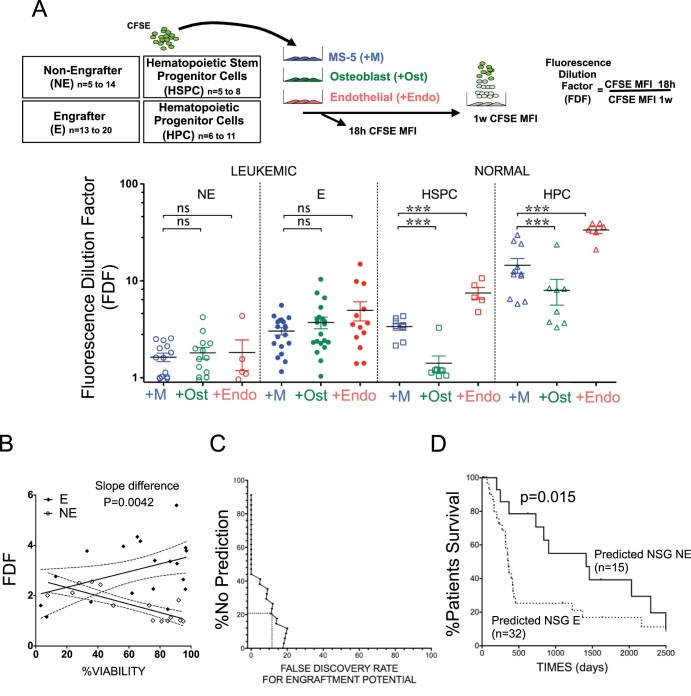
Ex vivo AML sample proliferation measure used to predict patient derived xenograft failure. **(A)** NE samples have a lower FDF compared with E samples independently of the co-culture system used. In contrast, normal HSPCs or HPCs are influenced by external cues affecting their proliferation profile. The upper flow chart illustrates the experimental design. Five to 14 different NE, 13 to 20 E, 5 to 8 HSPC, and 6 to 11 HPC samples stained with carboxyfluorescein succinimidyl ester were either co-cultured for 1 week with mesenchymal MS-5 cells (+M) or osteoblastic SaOS-2 cells (+Ost) or endothelial HUVECs (+Endo). For each sample and condition, FDF was determined as the ratio of MFI 18 h after staining to MFI measured after 1 week of co-culture. **(B)** FDF and viability regression analysis for E and NE samples co-cultured for 1 week on MS-5. The 95% confidence band is displayed (*dashed curve*). **(C)** A training data set was generated from **(B)** to derive a computational model for predicting E and NE status for an unknown sample. The data indicate the observed false discovery rate of predictions against the percentage of samples for which the model makes no prediction. **(D)** Patient survival dichotomized by predicted NSG engraftment potential. A two-sided Wilcoxon test was applied. AML = acute myeloid leukemia; E = engrafter; FDF = fluorescence dilution factor; HUVECs = human umbilical vein endothelial cells; L-LTC-IC = leukemic long-term culture-initiating cells; NE = nonengrafter.

To investigate further the dynamics of leukemic stem/progenitors ex vivo, we next exploited the newly defined fluorescence dilution factor (FDF) using carboxyfluorescein succinimidyl ester (CFSE) staining to track cell division over 7 days [10]. This parameter, which has a value ≥1, measures the median dye dilution of CFSE for the nonapoptotic leukemic cell population. We previously found that the FDF strongly correlates with leukemic stem/progenitor enrichment in the sample and identified that NE samples have a lower FDF compared with E samples (*p* < 0.001). We further state here that the mean FDF values for the E and NE groups are not influenced by co-culture of the samples with human osteoblastic or endothelial cells. In contrast, sorted normal hematopoietic stem/progenitor cells (HSPCs) or normal hematopoietic progenitor cells (HPCs) clearly had a lower FDF when cultured with osteoblastic SaOS-2 cells and a higher FDF when cultured with endothelial human umbilical vein endothelial cells (HUVECs) as compared with the MS-5 co-culture system (Fig. 2A, *p* < 0.001). These new results suggest that primary AML expansion ex vivo is self-controlled and that its measurement can distinguish E and NE samples.

**Supplementary Figure E4:**
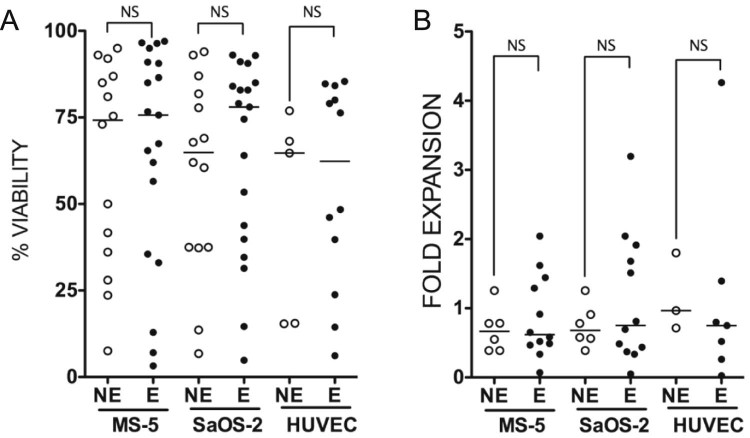
AML samples viability and proliferation in co-culture with MS-5 or SaOS-2 or HUVEC do not distinguish E and NE. **(A)** Data shown represents the NE and E AML cells percent viability after 1-week co-culture with MS-5, SaOS-2 or HUVEC assessed by DAPI and Annexin V exclusion (NE, E on MS-5 n = 14, 19. NE, E on SaOS-2 n = 14, 19. NE, E on HUVEC n = 5, 12). **(B)** Data shown represents AML fold expansion determined by FACS (NE, E on MS-5 n = 14, 19. NE, E on SaOS-2 n = 14, 19. NE, E on HUVEC n = 5, 12). Each symbol represents a single patient sample cultured in triplicate wells. Comparisons were made using a paired Student's t-test.

Lastly, we wonder if the CFSE-based assay could be used to predict xenograft success. When FDF was plotted as a function of total leukemic cell viability determined on day 7, we discovered a distinct pattern between 19 E and 14 NE samples. In the NE samples, lower proliferation was associated with lower viability, and contrarily, in the E samples, increased proliferation was associated with higher viability (Fig. 2B). Of note, the viability after 1 week or the fold expansion determined by the cell count did not differentiate E and NE samples regardless of the co-culture system used (Supplementary Figure E4, online only, available at www.exphem.org), which further indicates the utility of FDF as a predictive variable. With use of the observed data, a logistic regression model was constructed and used to generate a single predictive parameter (*Ɵ*) for any given data set, with prediction thresholds calculated from the observed data:

$$\theta =\frac{\exp\left[\begin{array}{c} \left(3.96\text{E-}02\times \text{viability}\times \text{FDF}\right)\\ -\left(7.09\text{E-}02\times \text{viability}\right)-7.09\text{E-}02 \end{array}\right]}{\left\{1+\exp\left[\begin{array}{c} \left(3.96\text{E-}02\times \text{viability}\times \text{FDF}\right)\\ -\left(7.09\text{E-}02\times \text{viability}\right)-7.09\text{E-}02 \end{array}\right]\right\}}$$

The nonengrafter prediction tool implementing this equation in Excel is available in Supplementary Table E2 (online only, available at www.exphem.org). If θ > 0.6363636 then the sample status is predicted to be “engrafter.” If θ < 0.3535354, then status is predicted to be “nonengrafter.” If 0.3535354 ≤ θ ≤ 0.6363636, then *no* prediction is made. For this training cohort, this modeling forecast engraftment status for 80% of the cases with 90% exactness independent of prognostic group (Fig. 2C). To validate our modeling, we wonder if this engraftment prediction would be as efficient as the actual tested patient derived xenograft status in predicting patient outcome (illustrated in Supplementary Figure E2A). FDF and viability after 1 week were determined on another cohort of 53 patient samples. A prediction was made using the above equation at the 0.05 FDR level for 70% of the sample. No prediction could be made for 16 samples. For the remaining samples, the observed patient survival times, upon the E and NE predictions, resulted in a significant difference in median survival (*p* < 0.026 level) (two-sided Wilcoxon test, Fig. 2D). This result is evidences of the reliability of our prediction calculator in stratifying AML aggressiveness. Our results indicate that the initial size of the leukemic stem/progenitor compartment, as well as the ex vivo cycling behavior, correlates with the xenograft potential of the sample. Thus, the actual stemness of the cycling subpopulation identified ex vivo should be further investigated, notably through their transcriptome analysis using the leukemia stem cell gene signature defined recently 20, 21. This could resolve whether the divisions observed are related to self-renewal potential. Alternatively, good outcome-related NE samples might also undergo more differentiation than poor outcome-related E samples. However, based on our recent analysis, using similar ex vivo culture conditions, we did not observe any increase in differentiation markers [22]. Furthermore, the fact that NE could engraft in NSG mice by extending the observation period (Fig. 1D) [6] suggests that these samples are not simply more differentiated than E samples.

Although a successful xenograft can be anticipated from poor-prognosis and good-prognosis patient samples, our technique should be especially helpful in including intermediate-risk samples, which represent the majority of samples, and in maintaining the standard experimental duration at 12 weeks. Furthermore, among the 3Rs Principles of Humane Experimental Technique by Russell and Burch (replacement, reduction, and refinement) our methods and the *Ɵ* value should serve for the reduction and refinement principles, by minimizing the number of animals used for preselecting E samples and by avoiding unnecessary failed xenografts with NE samples.

## Figures and Tables

**Supplementary Table E1:** Characteristics of AML patient samples

Patient ID	age	sex	WBC (G/L)	FAB	Karyotype	CD34+ (%)	CD34+/CD38− (%)	1 SL-IC per injected cell number		1 L-LTC-IC per AML MNC number plated at T0
								in NS	in NSG	
**Non Engrafters**										
1	65	M	112.8	AML M4	INV (16)	61.28	2.38	*NA	*†NA	67,969
2	60	F	74	AML M4	NORMAL	11.132	0.132	*NA	*NA	-
3	56	F	183.7	AML M4	TRISOMY 13	0.171	0.028	-	*NA	30,875
4	50	F	10	AML M2	NORMAL	2.478	0.528	*NA	*NA	663
5	66	F	6.1	AML M1	NORMAL	4.662	3.77	*NA	*NA	76,984
6	37	M	62.6	AML M0	19	84	10	*NA	*NA	21,937
7	66	M	6.3	AML M4	INV (16)	53.201	0.701	-	*†NA	81,511
8	28	M	296.7	AML M4	NORMAL	2.899	0.589	-	*NA	-
9	46	M	32.8	AML M4	INV (16)	65.64	6.74	-	*NA	4,565
10	24	M	180	AML M1	DEL(5)	77.888	0.188	-	*NA	-
11	22	F	70	AML M1	t(8;21)	79.573	0.973	-	*†NA	-
12	68	M	29.5	AML M2Eo	T(8;21)			-	*NA	-
13	59	F	61	AML M4	INV (16)	67.5	12.4	*NA	*†NA	11,996
14	46	M	1.1	AML M2	7Q-	2.265	0.995	-	*NA	-
15	57	M	4.6	AML M4	NORMAL	7.3	3.33	-	*†NA	-
16	28	M	160	AML M5	t(11;19)(q23;p13.1)	4.125	0.905	-	*NA	-
17	23	M	20.4	BI-PHENOTYPIC	+13, T(3;14)	57.353	0.353	-	*NA	8,519
18	83	M	85	AML M4	NORMAL	0.267	0.159	*NA	*NA	2,481
**Engrafters**										
19	43	F	71	2ndary AML	11P-,+CLONAL T(2;3)	0.226	0.01	2,702,541	1 875 795†	-
20	64	F	67.5	AML M4	TRISOMY12 + TRISOMY 21	88.6	3	*NA	*	-
21	73	M	27.7	AML M2	+11, +13	23.7	11.7	*	*	-
22	57	M	11.9	AML M2	NORMAL	4.355	0.785	13 650	348‡	11,441
23	17	M	32.5	AML M2	NORMAL	48.75	1.15	*	*‡	
24	35	M	71.4	AML M4	+3, +10	1.232	0.266	*	*	83.8
25	54	F	193.9	AML M5	+1	2.924	0.914	-	*	
26	59	M	-	AML M5	-	10.59	8.58	*	*	1934
27	55	F	175.7	AML M4	NORMAL	2.739	1.96	-	*	-
28	15	F	111.7	AML M5	NORMAL	2.96	1.38	26 341 443	4 647 288	-
29	37	F	100	AML M4	NORMAL	77.033	0.433	2 451 269	421 968	-
30	69	M	98.7	AML M1	NORMAL	0.99	0.679	*	*‡	213
31	26	M	142.3	AML M1	NORMAL	48.117	0.617	2,223,827	43413‡	764
32	63	F	94.3	AML M1	FAIL	2.219	0.329	9071496	5252551	
33	53	F	9.6	AML M2	NORMAL	3.054	0.214	*	*	5078
34	47	M	313.8	BI-PHENOTYPIC	NORMAL	14.55	6.32	78108	2876‡	134
35	46	M	165.2	AML M4	11Q23	89.4	19.3	2229695	657274‡	3.69
36	56	M	42.9	AML M4	NORMAL	21.62	3.52	*	*	7948
37	57	M	3.4	2ndary AML	COMPLEX	48.51	2.81	333,699	3007‡	35.5
38	44	F	44.1	AML M5	NORMAL	0.117	0.03	-	*	-
39	67	M	126.7	AML M1	13	0.495	0.213	*NA	*	55
40	17	M	162.1	AML M5	ins(10;11)(p12;q23q21)	85.09	1.19	7663450	2 020 413†‡	-
41	65	M	19.5	AML M2	add(3)(q1),add(5)(q13),add(7)(q22),+8,add(11)(p15)	74.4	38.3	*	*	-
42	46	M	31.3	AML M2	NORMAL	0.0194	0.0073	-	*	-
43	66	M	2.2	AML M1	TRISOMY 13			-	*	11,796
44	72	F	116.8	AML M2	NORMAL	0.34	0.185	175 966	2 028‡	95.6
45	62	M	29.1	AML M2	COMPLEX	0.0569	0.006	5 518	448‡	-

NSG Mice were injected with 10.7 T depleted peripheral blood nucleated cells from the peripheral blood of AML patients. Murine marrows were analyzed 12 weeks after transplantation for the presence of human hematopoietic cells. AML Engraftters were defined as samples capable to establish the maintenance of human CD33+/CD45+ myeloid cells without an accompanying CD19+/CD45+ B-cell population (sample #19 to #45). All samples were obtained at diagnosis. WBC: White Blood Cell Count. FAB: French-American-British classification. Not determined (-).

* Tested for engraftment in the model.

† Engraftment potential tested in NSG new born. NA: tested with a negative assessment results.

‡ For some engrafters expressing or non expressing CD34 or CD38 cell populations were sorted and tested for engraftment potential as displayed in suplemental figureX.

**Supplementary Table E2:**
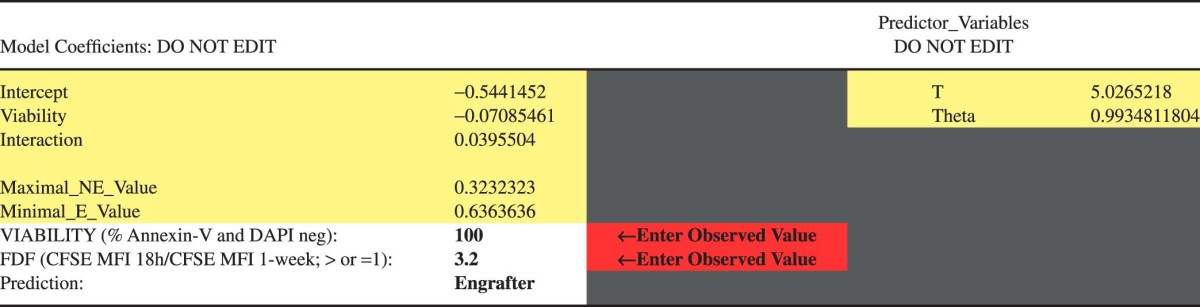
Xenograft prediction tool
